# Narrative Abilities and Episodic Memory in School-Aged Children Followed by Child Protective Services

**DOI:** 10.3390/children8100849

**Published:** 2021-09-26

**Authors:** Sanmya Salomão, Catarina Canário, Orlanda Cruz

**Affiliations:** Faculty of Psychology and Educational Science of the University of Porto, 4200-135 Porto, Portugal; anacanario@fpce.up.pt (C.C.); orlanda@fpce.up.pt (O.C.)

**Keywords:** school-aged children, narrative abilities, event schema, narrative coherence, narrative temporal cohesion, episodic memory, child protective services, disruptive behaviours, exposure to domestic violence, neglect and abuse

## Abstract

The ability to narrate routine familiar events develops gradually during middle childhood, in increasingly higher levels of coherence and temporal cohesion. Improvements in episodic memory are also observed, reflecting children’s increasing ability to recall specific circumstances of past events and personal experiences. Even though several studies have evaluated children’s narrative abilities and episodic memory, little information is available regarding the children exposed to risks that justify their referral to Child Protective Services (CPS). The current study analysed children’s narrative abilities and episodic memory performance, according to the circumstances related to the referral to CPS. Event schema representation, narrative coherence, narrative temporal cohesion, and episodic memory concerning routine and specific personal events in family context were analysed in a sample of 56 school-aged children followed by the CPS in Portugal. Children referred to CPS due to disruptive behaviour presented higher episodic memory performance, compared to those exposed to domestic violence, neglect, and abuse. No significant differences were found between groups regarding narrative abilities related to familiar routine events. Results highlight the relevance of evaluating the adverse circumstances that lead to CPS referral, considering the levels of risk and danger involved, given its differential effects on children’s episodic memory development.

## 1. Introduction

### 1.1. Parental Maltreatment and Detrimental Effects on Children’s Narrative and Memory Abilities

Parental maltreatment is increasingly recognised as a worldwide concern, given the lifelong damages caused to a child’s mental health and the intergenerational dynamic perpetration of the problem in vulnerable families. By definition, parental maltreatment refers to actions that result in (potential) physical or psychological harm to the child, performed in different levels, varying from threats to direct or indirect acts, affecting the child’s well-being and violating their fundamental rights [1]. In this broad definition are included diverse parental practices, family situations and settings in which the harm results from an intentional and deliberate act, as well as omission, neglect, abandon, or lack of appropriate child care. Traditionally, four major categories of parental maltreatment have been proposed: physical abuse, sexual abuse, neglect, and emotional maltreatment, involving diverse experiences that should be distinguished and evaluated according to the level of risk and potential harm, severity, and specific implications [1,2].

It has been demonstrated that parental maltreatment negatively affects key aspects of child development, such as emotional regulation, executive functioning, internal working models, and overall adjustment [3,4]. Regarding temporal cognition skills, it has been demonstrated that abused and neglected children underperformed in tasks that demanded basic abilities to order events and define adequate temporal sequences [5,6].

Beyond language impairment, child maltreatment also may compromise children’s ability to provide an adequate narrative account of their experiences [7]. Valentino and colleagues evaluated autobiographical memory among abused, neglected, and non-maltreated school-aged children, showing evidence that childhood maltreatment may be associated with memory impairment, in particular for specific episodes in children’s personal past [8]. In contrast with the results found in the developmental research of memory skills with non-maltreated children, in a sample of school-aged participants exposed to family violence, neglect, and maltreatment, older children recalled fewer specific memories than younger children [9].

Recent developments in the field of Child Protection have led to a renewed interest in taking the children’s perspective into account. Listening to children is a challenging task and many aspects should be observed, for instance, the child’s capacity to recover information and to elaborate narratives about personal past experiences [10].

### 1.2. Narrative of Familiar Routine Events: Event Schema Representation, Narrative Coherence and Narrative Temporal Cohesion

Narrative improvements are observed since early developmental stages when children talk about familiar routine events, reporting recurrent everyday activities [11]. An event is defined as a self-involving situation in which someone engages in purposeful activities, interacting with other people, taking part in ongoing happenings, observing, or acting to achieve some result [12]. Event schemas (or scripts) are structured representations that encompass the essential elements of the situation reported as an event—the actors involved, the action, the context (time and place), the circumstances and the unfolding happenings [13]. The development of event schemas depends on the exposure to experience and familiarity with previous events, but also on the child’s developing competence to grasp causal and temporal relations between the events [14], and to segment distinct actions, depicted from the continuous event flow [15].

Event schema representations also depend on the organised temporal sequence of actions, providing structure to the narrative and establishing the event circumstances [16]. The routines of daily life display an opportunity to perform and enhance event schemas, based on the regular sequence of actions related to social patterns, the framed roles played by actors and the expected flow of events that follow certain scripts. Studies have yielded evidence that children develop their event schemas and their ability to understand more complex structures and flexible representation of events around the preschool and early school years [17]. Around 5- or 6-years old children accomplish organized narratives about regular familiar events, based on well-established scripts or schemas [18], and a coordinated temporal sequence of events [19,20].

Very early in their development, children can produce reasonable and reliable narratives about the past, especially if they are talking about familiar and personal events [10,11,21,22]. In middle childhood, it is expected that children’s narratives accomplish basic coherence, even though they continue to develop along this period and through adolescence [23]. Normative non-maltreated school-age children can provide narratives with the appropriate contextual and referential information to localize the events in a general spatial-temporal frame, also including evaluative elements that convey the meaning of the reported events [24,25]. At this stage of development, the child’s narratives are relatively complete in terms of the variety of specific details provided, including basic information that orient the appropriate comprehension of what happened (actions and activities), who was involved, where and when the event took place and also how and why it happened (i.e., some descriptive and evaluative information) [26]. Altogether, these attributes define the narrative coherence.

The debate about narrative development has gained fresh prominence with many arguing that narrative temporal cohesion, besides narrative coherence, is an essential dimension to narrative analysis [27,28,29,30]. Narrative temporal cohesion regards the dynamic temporal relation between the events, contributing to the integration of the narrative elements in a flow of information that makes sense to the listener [31]. Reese and colleagues observed a dramatic development comparing children from 6- to 11-years old, where the younger participants were able to provide only some information about time and/or place, and the older (school-aged) children achieved the ability to specify time and place of events in their narratives [29]. In addition to the time context references, temporal linking terms are essential features of a cohesive narrative. Fivush and colleagues distinguished simple temporal markers, that describe basic sequencing of events and successive actions, from complex ones, that establish conditional, alternative, comparative, and simultaneity relations between the events [29]. During middle childhood, narrative temporal cohesion improves significantly, with the use of more complex temporal connectives and more descriptive information. Compared to pre-school children, school-aged children go beyond providing basic information about time and place in their narratives, moving from simple sequence to more complex temporal relations among events, expressing simultaneity, conditional, and alternative relations, chaining events both causally and sequentially [28,29,30,31].

There is evidence of significant development of event schema representation and narrative coherence in middle childhood, with improvements in narrative temporal cohesion as well [31]. Especially, the narrative of familiar routine events becomes more elaborate, complex, coherent, and temporally organized [16]. Studies have demonstrated that, along school years, children report more component activities related to each event, more optional and conditional actions, with an increasing integration between routine events [10,32].

### 1.3. Narrative of Specific Personal Past Events: Episodic Memory

Episodic memory regards the narrative of single past events in a specific spatial-temporal context, evoking the recall of the event circumstances from where the first-hand information can be recovered (or re-constructed), and shaped by personal experience [33,34]. This specific memory system is defined as an aspect of explicit memory that entails the recall of an event or information all together with its original circumstances and particular contextual details, allowing one to re-experience past events [35]. In other words, “the essence of episodic memory lies in the process of recollection, by which one can not only reinstate the contextual details of an event but can also mentally re-experience it” [35] (p. 1046).

Episodic memory is a hypothetical memory system that allows people to consciously re-experience past experiences. Along with semantic memory, it is part of the declarative (explicit) memory system which is “accessible to consciousness and concerns the ability to remember the past (episodic memory) and to extract meaning and regularities from those remembered experiences (semantic memory)” [36] (p. 205). Episodic memory refers to the recall of unique events, delimitated by specific circumstances (place and time) of that singular event occurrence. Three key aspects are encompassed in the concept of episodic memory: a sense of self, an autonoetic consciousness awareness, and a subjective sense of time. Together, these properties enable what is called mental time travel, transporting oneself across time to mentally relive a past episode, recovering the subjective experience of a past event [33].

Episodic memory develops gradually along middle childhood and adolescence [37,38]. Studies have also demonstrated the effect of age in episodic memory performance, especially in cued tasks, in which children are prompted to talk about personal specific episodes [39,40]. Main age effects were observed on episodic memory, with increasing abilities analysed in normative samples of children from 6- to 12- years old [41]. Several studies, thus, have evidenced that narrative abilities are strongly correlated with episodic memory performance [42,43,44].

### 1.4. The Current Study

Children and families followed by Child Protective Services (CPS) are exposed to diverse vulnerable situations, demanding an appropriate approach, sensible to particular settings. Exploring the particularities of different adverse circumstances that lead to CPS referral is crucial in the identification of possible levels of risk for children’s well-being and mental health.

According to the Law of Protection of Children and Young People at Risk, Law 147/99 [45], the Portuguese CPS assumes dangerous and risk situations when children are (a) abandoned, (b) subject to physical, psychological, or sexual abuse, (c) fail to receive the proper care and protection, (d) submitted to excessive activities or work that are inappropriate to their age, dignity and personal situation or harmful to their development, (e) directly or indirectly, subject to behaviours that severely affects their safety or emotional well-being, and (f) engaged in activities or show behaviours that affect their health, safety, educational and developmental conditions, without their parents or legal representant objecting to protect and to come across the dangerous situation. Based on these concepts, the Portuguese CPS adopts a classification system that includes several categories considered as reasons for the application of protective measures: (a) neglect, (b) domestic violence, (c) disruptive behaviour, (d) lack of educational rights, (e) physical maltreatment, (f) psychological maltreatment, (g) child work exploitation, (h) abandon, and (i) sexual abuse (further details on the classification are described in the Portuguese CPS annual report) [46].

The categories of reasons for referral to CPS observed in the present study were related to children’s disruptive behaviour, exposure to domestic violence, neglect, and abuse. The circumstances that lead to CPS referral due to children´s disruptive behaviour encompass severe externalizing behaviours that may cause potential harm to the child, anti-social actions, illegal activities, severe indiscipline, and maladjustment in social contexts, with most cases being reported by school professionals and not identified by the parents in a family context. Exposure to domestic violence refers to the circumstances where children witnessed physical or psychological violence within the family context. Children referred for neglect are exposed to behaviours that can represent a risk for their well-being, given the lack of proper supervision, parental care, and protection. Finally, the abuse category includes physical maltreatment and abusive use of parental authority, threatening to children´s integrity and well-being.

This study attempts to emphasize the children´s perspective by promoting their narrative of personal experiences in contexts of families at-risk and referred to CPS. The major interest of this study was on children’s narratives about familiar routine events and their episodic memory for singular experiences in the context of parent-child interactions in everyday life, exploring the particularities of different adverse circumstances that entail the referral to CPS.

Families at risk for breakdown that are followed by CPS usually adopt inconsistent and coercive parental practices, evidencing some difficulties in planning and maintaining a regular organization of daily routines [47]. The lack of regularity observed in neglectful and abusive contexts provides an inconsistent temporal frame and may bring detrimental effects on narrative abilities and episodic memory [5,6,7].

The purpose of the current study is to explore the effects of the circumstances of referral to CPS that place families at-risk for breakdown on children´s narrative abilities and episodic memory, evaluated through children’s narrative of familiar routine and specific personal events. Narrative abilities encompass the representation of event schema, narrative coherence, and narrative temporal cohesion. Episodic memory was evaluated through children’s narratives of specific personal events considering the parental practices described from their perspective.

Furthermore, the possible confounding effects of age as a covariate are addressed, particularly, regarding the developmental aspects and their possible effects on narrative abilities and episodic memory performance.

## 2. Materials and Methods

### 2.1. Participants

Fifty-six children aged between 6 and 12 years (60.7% boys, *M* = 9.53 years, *SD* = 1.61) took part in the study. Participants were recruited from vulnerable families followed by CPS in several cities of the northern area of Portugal due to adverse situations, being at-risk for family breakdown. All families were followed by CPS and referred to parenting support.

Fourteen children lived with their original family (25%) and most of the participants lived in other family arrangements: 30.4% with reconstituted families (*n* = 17) and 26.8% with single parents (*n* = 15), especially with mothers (*n* = 14). Nearly half of children’s parents were unemployed (*n* = 29) and 91.1% received some kind of social insurance and state financial support (*n* = 51). Thirty-eight participants lived in an urban area (67.9%) and half of the families have been previously referred to CPS (*n* = 28).

The CPS professionals provided information about the family, including reasons for current referrals, the protective actions applied and also about the risks and the effective harm involved. Considering the reasons for referral in this sample, classified according to the definition adopted by the Portuguese CPS [46], participants were divided into three groups: (1) children who were followed due to disruptive behaviour, most cases reported by school professionals (*n* = 16), (2) children exposed to domestic violence (*n* = 17), and (3) children exposed to neglect and abuse (*n* = 21). The third group includes children referred to CPS due to neglect (*n* = 19) and due to physical maltreatment (*n* = 4) based on the similarity of their circumstances, according to the information provided by the CPS professionals. The main demographic characteristics of children and their families according to the categories of reasons for referral are presented in Table 1.

In the category of children referred to CPS due to disruptive behaviour, most of the children (81%) were boys (*n* = 13) and most part (93.8%) lived in urban areas (*n* = 15). Just a small part (6.3%) lived with single parents (*n* = 1), in contrast with the majority (41.2%) of children referred for exposure to domestic violence (*n* = 7), and about a third (30.4%) of children referred for neglect and abuse (*n* = 7), who lived with single parents.

### 2.2. Measures

#### 2.2.1. Narrative Abilities

Event schema, narrative coherence and narrative temporal cohesion were observed through the participant’s narrative of familiar routine events. Similar to the procedure used in previous studies [48], children were asked to provide the report of a typical day (the regular activities described by children as part of their daily routine), since they wake up, until the time to go to bed at night, trying to remember all the activities they were involved in, including as many details as possible to describe exactly what happened in the previous day or the last typical day the child could remember.

The coding of children’s narrative of the familiar routine was based on the well-established procedure applied in several narrative studies [26,27,49]. First, the number of events generated to describe the daily routine (i.e., the representation of event schema) was analysed evidencing the structure of the daily script. The activities described by the children were assembled in nine exhaustive categories of events that might convey the regular routine of a typical school day: (1) waking up, (2) having breakfast, (3) getting ready for school, (4) attending school, (5) having lunch, (6) structured extra-curricular activities, (7) free time, non-structured activities, (8) having dinner, and (9) going to sleep. Since the interest of the study was on the representation of event schema in the report of a typical day, exceptional activities described by the child were not included in the categories above mentioned. The total event schema score was obtained by an account of the total of different event categories presented (maximum of nine).

For the analysis of narrative coherence, each narrative was segmented in propositions, that is, independent clauses including a subject and predicate. Then eight categories (see Table 2) were used to describe the kind of information provided in the children’s report of events: action, object, people, place, time, justification, description and evaluation. The total narrative coherence score was obtained by summing up the number of different categories represented (maximum of eight).

The narrative temporal cohesion was analysed, considering the sequence and the temporal markers observed that convey chronological relations between the events. Two categories of temporal markers were observed: (1) simple temporal markers, such as then, first, next, before and after, and (2) complex temporal markers, that indicate simultaneity, alternative, conditional and temporal relations between events, like while, when, meanwhile, sometimes and usually. The total narrative temporal cohesion score was obtained after applying the following scale: (0) total absence of temporal markers and temporal sequence, (1) absence of temporal markers, but the events presented in a minimally cohesive temporal sequence, (2) presence of simple temporal markers, only, (3) presence of complex temporal markers, only, and (4) presence of simple and complex temporal markers.

#### 2.2.2. Episodic Memory

To measure episodic memory ability, an adapted version of the Episodic Thinking Interview was conducted, including a cued technique to examine episodic memory in school-aged children (i.e., the ability to recall detailed specific personal events) [50]. To highlight the child’s perspective about parental practices, three specific interactive situations of familiar routines were used as cues. Children were asked to describe what usually happens in three situations, answering the questions: “What do you like to do together with your mother/father?”, “What does your mother/father do when you behave well and do something really nice?”, and “What does your mother/father do when you break the rules and misbehave?” After asking the child to report what usually happens in each situation, we immediately prompt the child to recall a specific event to illustrate that scenario, describing the personal past event in as much detail as they could remember: “Describe, out loud and with as much detail as possible, what happened in one specific time you remember when you and your mother/father did ___ together”, “when you behave well, doing something really nice and your mother/father did ___”, and finally “when you broke the rules or misbehave and your mother/father did ___”.

A scoring system adapted from previous studies was used to analyse the episodic memory performance in the narrative of specific personal past events [26,50,51]. Episodic memory scores ranged from 1 to 5 and considered the episodic nature and specificity of the event (single or repeated), the spatial-temporal information that allows the definition of event circumstances (the context where and when the event occurred), and the presence of phenomenological details that entails how the participants felt, what they thought, their impressions and other images associated with the experience recovery (see Table 3).

### 2.3. Procedure

Each child’s data was collected in a single session that took place in the community-based services attended by the presently constituted family. The data collection procedure was implemented by a research team member, for approximately 20 min and consisted of an interview about familiar routines and specific personal events reported by the children.

A safe and secure environment was created to minimise the discomfort and to encourage the child to talk about family routines and parental practices. Given the sensitive nature of some interview questions, participants and their parents were told that the content of the interview would be kept confidential unless the child reported any violation of her rights or any abusive situation.

All children’s families provided informed consent and signed release forms authorizing the child’s participation in the study, with verbal assent from the children. The current study is part of a wider research project to evaluate the effectiveness of an evidence-based parenting intervention delivered in community-based settings to vulnerable families, at-risk for family breakdown (further details on the project are described elsewhere) [52]. The research project from which the current study stems from received ethical approval from the Ethics Committee of the Faculty of Psychology and Education Science at the University of Porto (approved 14 April 2020, ref: 2020/04-2), and also approval from the Data Protection Unit of the University of Porto (approved 3 September 2019, ref: 2018091915006231).

The interviews were audio-recorded, transcribed and coded according to the different analysis strategies applied to the narrative of routine and specific events. Interrater agreement was assessed on 20% of the narratives analysed, after the codification by two independent ratters. For the event schema and narrative coherence, defined as continuous variables, interclass correlation was estimated. For the narrative temporal cohesion and episodic memory, defined as categorical/nominal variables, Cohen’s kappa was used to estimate inter-coder reliability. The interclass correlation coefficient for event schema was .97, ranging from .90 to .99, and for narrative coherence, the interclass correlation coefficient was .95, ranging from .81 to .99. For narrative temporal cohesion, kappa was .60, with 87.5% of agreement, and for the three episodic memory singular scores, kappa was .60, .59 and .69, with 70% of agreement, considered moderate to substantial agreement [53].

### 2.4. Analytic Plan

Analyses of covariance (ANCOVA) were performed using the software IBM SPSS v.27 [54]. The models (one per outcome variable) included each dependent variable, the categories of referral to the CPS as the between factor subject. A Bonferroni posthoc test was used in each analysis to compare mean differences across groups. According to a priori power analysis, performed using the software G*Power [55,56], a sample size with 56 observations would be enough to reach high effect sizes (effect size *f* = 0.50) considering *p* < .05, 95% power, one dependent variable, three groups, and one covariate.

## 3. Results

### 3.1. Preliminary Analysis

The descriptive statistics and bivariate correlations regarding the study variables are presented in Table 4.

The results provided evidence for strong positive correlations between the three measures of narrative abilities, event schema, narrative coherence, and narrative temporal cohesion. Likewise, moderate positive correlations were found between episodic memory and the three measures of narrative abilities. Children’s age was correlated with all the outcome measures (in a moderate or strong relation), and for this reason, it was included as a covariate in the analysis of variance performed.

### 3.2. Analyses of Covariance of the Effect of Categories of Reasons for Referral to CPS on Narrative Abilities and Episodic Memory, Controlling for Children’s Age

As presented in Table 5, the outcomes for event schema, narrative coherence and narrative temporal cohesion were not different according to the categories of reasons for referral to CPS, when controlling for children’s age. In fact, the outcomes were different for episodic memory, according to the categories of referral and controlling for children’s age.

**Figure 1:**
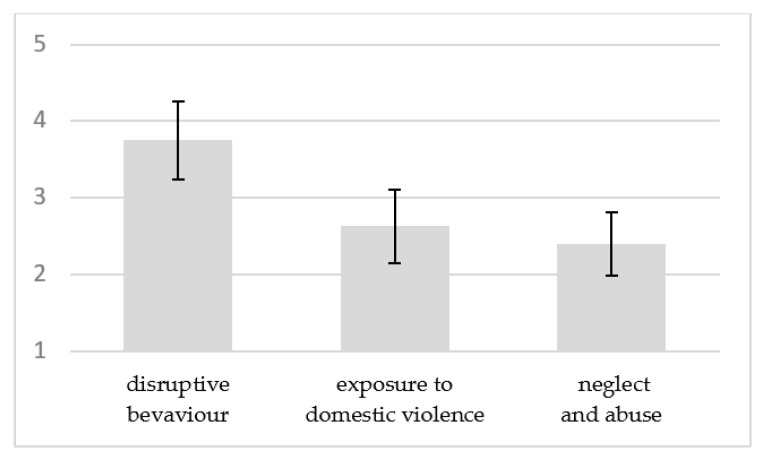
Episodic memory means according to the reasons for referral to CPS, controlling for children’s age.

As depicted in Figure 1, children referred to CPS for exposure to domestic violence, neglect, and abuse had significantly lower episodic memory performance, compared to children referred for disruptive behaviour. Bonferroni posthoc tests indicated that children referred to CPS for exposure to domestic violence (*M* = 2.55, *SD* = 0.23) and for neglect and abuse (*M* = 2.46, *SD* = 0.19) produced narratives with significantly poorer episodic memory performance when compared to children whose reason for referral to CPS was disruptive behaviour (*M* = 3.76, *SD* = 0.24). No differences in episodic memory performance were found between the other two groups.

## 4. Discussion

The present study was designed to explore the effects of the circumstances of referral to CPS on children´s narrative abilities and episodic memory, including the analysis of possible confounding effects of age, given the developmental aspects involved.

Event schema, narrative coherence and narrative temporal cohesion were intercorrelated and this result is consistent with the conceptual approaches that consider these abilities as integrated dimensions, representing basic elements and properties of narrative production [17,18]. The strong positive correlation observed between the narrative measures analysed in this study provides some support for the conceptual premise that the narrative of familiar routine events entails aspects of structure, coherence and temporal cohesion, as fundamental dimensions to be observed [27,28,29].

A positive moderate to strong correlation was also observed between children’s age, event schema, narrative coherence, narrative temporal cohesion confirming the developmental trends already described in the literature [11,12,33,34,35,36]. This finding corroborates previous studies that have demonstrated evidence of increasing narrative abilities observed during middle childhood (i.e., event schema representation) [14,15,16], narrative temporal cohesion [30,31,32], and narrative coherence [23,24,25,26]. Episodic memory was also correlated with age, corroborating previous studies describing an age effect in episodic memory during middle childhood [37,38,39,40,41,42,43,44]. The positive strong correlation observed between episodic memory, event schema, narrative coherence, and narrative temporal cohesion is also in line with the literature [42,43,44].

When comparing event schema, narrative coherence and narrative temporal cohesion scores according to different reasons for referral to CPS and after controlling for children´s age, no significant differences were observed. In contrast, children´s episodic memory performance was found to differ across the different categories of reasons for referral, with children referred to CPS due to exposure to domestic violence, and neglect and abuse presenting lower scores compared to children referred for disruptive behaviour.

The three categories analysed as reasons for referral to CPS represent adverse conditions that affect children´s narrative abilities [7] and episodic memory [8,9] and their regular developmental trend described in the literature for normative samples [11,29,30,31]. However, each category may have different effects, depending on the specific circumstance entailed. Describing particular characteristics of each category of reasons for referral to CPS, according to the classification system adopted by the Portuguese CPS [46], would help to explain the results observed in the present study, after group comparison.

A large range of situations is included in the category of disruptive behaviour as a reason for referral to the Portuguese CPS, varying from externalizing behaviours, anti-social or illegal actions to maladjustment in social contexts that may represent harm and potential risks to the child. Most cases of disruptive behaviour are reported by school professionals and are not recognized by the parents in the family context, eventually contributing to inappropriate parental practices in terms of child care and protection. Therefore, it is possible to discuss if the referral to CPS based on disruptive behaviour isn´t also related, at some level, to neglect or at least to the lack of adequate parental supervision. Results obtained from the present study reveal that more than half of children referred to CPS due to disruptive behaviours (56.3%) have had previous referrals.

On the other hand, exposure to domestic violence regards specific situations where children witnessed physical or psychological violence within the family context, and were exposed to potential harm related to violence. The third category defined in the present study was neglect and abuse, referring respectively to situations that represent severe risks for children’s safety, integrity, and emotional well-being, given the lack (or inadequacy) of parental supervision and abusive parental practices. In both categories of referrals to CPS, exposure to domestic violence, and neglect and abuse, possible detrimental effects can be observed, directly affecting parent-child relations, with severe impacts on the relational context of everyday family life.

Referrals related to disruptive behaviour represent a risk since children get exposed to potentially harmful situations that can also have implications for the future. However, compared to the other reasons for referral, it can be assumed that the category of disruptive behaviour represents a lower level of risk and a less harmful situation.

The present study findings suggest that considering episodic memory abilities in the narrative of personal past events, exposure to domestic violence and neglect and abuse seem to represent more serious adverse circumstances, possibly involving higher levels of risk and more severe impacts when compared to the referrals to CPS due to disruptive behaviour. In contrast to these findings, no effects were observed in narrative abilities applied in the narration of familiar routine events. A possible explanation for these results may be the fact that event schema, narrative coherence, and narrative temporal cohesion are already established elements of narrative at this stage of development, which makes them less prone to the differential effects of more or less adverse circumstances. The narrative skills concerning basic orientation and reference elements related to narrative coherence emerge in the early stages of child development and are established in school-age children, especially in the narrative of familiar events [17,18,22,29,30,31].

Another possible explanation for this result is the specific nature of narratives about familiar routine events. In this narrative category, children talk about recurrent everyday activities and may use scripts and impersonal speech in their report, supported by established event schema representations that convey structure and enhance narrative coherence and narrative temporal cohesion [13,14,15,16,17,18,19,20,21,22]. In contrast, the narrative of specific personal past events establishes additional challenges and entails episodic memory abilities [35,36]. Episodic memory requires a sense of self and a subjective sense of time, providing the recall of a personal episode, not only with reference to specific events but also including the original circumstances and particular contextual details involved in the subjective experience [33,34]. Compared to the narrative of routine events, the mechanisms of episodic memory abilities involved in narratives of personal experiences are more complex [37,38,39,40,41] and possibly more sensitive to differential adverse influences, according to their lower or higher levels of risk. This is in line with the results of the present study, which show a significant difference in children’s episodic memory performance, according to the reasons and circumstances that motivate the referral of children and their families to CPS, after controlling for age effects.

Besides, the narrative of personal past events addresses directly the parental practices and interactions between children and their parents. It may be that in the groups of children referred to CPS due to exposure to domestic violence, and neglect and abuse, the narrative of personal events concerning parent-child interactions in the family context would be more sensible to impairment effects.

These results suggest that children referred to CPS due to disruptive behaviour may be in a less adverse situation, of less danger, compared to children exposed to domestic violence, and neglect and abuse. Previous studies have already discussed, specifically, the detrimental effects of parental maltreatment on children’s ability to provide narrative accounts of their experiences [7,8,9]. The major concept of parental maltreatment defined in the literature is broad and encompasses a series of different circumstances addressed in the context of Child Protection, involving deliberate abusive acts, neglect, or lack of appropriate child care [1]. Defining specific categories of maltreatment is challenging and crucial in the evaluation of parental practices and risks observed in each family context [2].

Particular categories that are able to describe in-depth the diversity of adverse circumstances should be distinguished and evaluated according to the risks they represent and their differential effects on a child’s well-being. In addition, specific family situations, including indicators of the levels of stability and safety experienced within the family environment should be explored in future studies, particularly its role as a moderator of the impact of vulnerability on children´s narrative abilities and episodic memory.

Future studies should address the cognitive development of children referred to CPS using prospective designs and evaluate the effects of the different reasons for CPS referral, the different levels of risk, risk and protective factors that children are exposed to, family-specific characteristics, the duration and existence of previous referrals, the prevalence of the exposure to the aggressor (in cases of violence or abuse), the age of the child exposed to neglect and abuse, and the effects of protective measures applied.

The present study provides additional evidence of detrimental effects of exposure to domestic violence, and neglect and abuse on a child’s episodic memory and the ability to report personal experiences from a subjective perspective [7,8,9]. The findings emphasize the child’s perspective and their account of personal events and experiences in the family environment, in contexts of disruptive behaviour, exposure to domestic violence, neglect and abuse, distinguishing the differential effects of the particular reasons why family and children are referred to CPS. The study follows a cross-sectional design and is of exploratory nature. Limitations include the relatively small sample size and some possible bias related to children’s different reasons for CPS referral. Data collection took place since October 2019 and was negatively affected by the COVID-19 pandemic. In an effort to contain the spread of the virus, several lockdowns were imposed by the Portuguese Government, and as a consequence the research team was precluded from evaluating the children face-to-face in community-based services for several months, limiting the sample size. Other limitations regard a possible bias related to the fact that the children referred due to disruptive behaviour can be exposed to fewer risk factors. Future studies should replicate the current study analysis in larger sample sizes, and further explore the role of cumulative risk factors when evaluating the effects of the reasons of referral to CPS on children’s narrative abilities and episodic memory.

The current study analysed the total episodic memory but did not address the episodic memory skills revealed in each one of the situations used as cues to evoke a child’s narrative. Future studies should explore the content of children’s narrative regarding parent-child interactions and family environment, addressing specific parenting practices described from children’s perspective, and their impact on children’s wellbeing. Further studies should also present a more refined analysis of each dimension in narrative coherence, especially regarding referential information that provides the contextual details of the narrative events, and the use of simple and complex temporal markers that convey narrative temporal cohesion.

## Figures and Tables

**Table 1:** Demographic characteristics by reasons for referral to CPS, presented in percentage (%), means and standard deviations (in parentheses).

	Disruptive Behaviour (*n* = 16)	Exposure to Domestic Violence (*n* = 17)	Neglect and Abuse (*n* = 23)
Children			
Age (years)	10.39 (1.42)	9.09 (1.45)	9.26 (1.68)
Grade level	4.19 (1.27)	3.18 (1.85)	3.35 (1.67)
Gender (male)	81.3%	47.1%	56.5%
Parents			
Years of schooling	7.25 (2.54)	8.18 (2.24)	7.04 (2.08)
Unemployed	43.8%	70.6%	43.5%
Families			
Single parents	6.3%	41.2%	30.4%
Original constitution	18.8%	23.5%	30.4%
Reconstituted	56.3%	11.8%	26.1%
Low-income	87.5%	94.1%	91.3%
Live in urban area	93.8%	64.7%	52.2%
Previous referral to CPS	56.3%	41.2%	52.2%

**Table 2:** Narrative categories used to code the narrative coherence ^1^.

Narrative Category	Tokens Whose Presence Was Coded
Action	Activities performed by the child
Object	Specific objects present and relevant to specify the activities described
People	Specific people or the gender or class of people present for or participating in the event
Place	Location of the event or a preposition indicating place
Time	Reference to time, including an indication of the order of specific events within an experience, placement of the event in time, or duration of an event
Justification	Justification or indication of causation (e.g., ‘‘because’’, ‘‘in order to’’)
Description	Adverb, adjective, other modifier, or prepositional phrase describing an action and how it was performed
Evaluation	Indication of personal evaluation of the event, for example, through use of an intensifier (e.g., ‘‘most’’, ‘‘biggest’’), intentional repetition (e.g., ‘‘I worked, worker worked and worked’’), use of a subjective modifier (e.g., ‘‘it was ugly’’), or mention of internal state (term conveying information about emotion, cognition, perception, or physiological state)

^1^ [26].

**Table 3:** Episodic memory coding system ^1^.

Score	Description	Examples
1	Absence of response, when the participant failed to report anything or reported general information only	*“We just play, that’s all”*
2	Reported of a vague event, with no contextual details, regarding time, space, or specific event circumstances	*“I behaved badly… hitting my sister and got grounded”*
3	Report of a generic event, that could be repeated or continuous, but situated in time and/or space, considering the event circumstances	*“We were in a trip to the beach one day and I behaved very well, we were playing in the car, then we had a good time there…the whole day”*
4	Report of a specific event (isolated and situated in time and/or space) without any phenomenological detail	*“Last week I went out, but I didn’t ask my parents if I could go to my cousin´s and when I came home later mom chided me...”*
5	Report of a specific event (isolated and situated in time and/or space) with phenomenological detail, such as imagery, emotions, or thoughts	*“Yesterday, I was doing homework while mom was in the kitchen. It was hard, but I did it all by myself and she was really happy and proud of me!”*

^1^ based on [26,50,51].

**Table 4:** Pearson (*r*) correlation between event schema, narrative coherence, narrative temporal cohesion, episodic memory (EM), and Children’s age, with Means (*M*) and Standard deviations (*SD*).

Measure	*M*	*SD*	1	2	3	4
1. Event schema	4.93	2.82	-			
2. Narrative coherence	4.93	1.88	.56 ***	-		
3. Narrative temporal cohesion	2.39	1.47	.63 ***	.67 ***	-	
4. Episodic memory	2.86	1.23	.46 ***	.42 **	.46 ***	-
5. Children’s age	9.54	1.62	.55 ***	.30 *	.42 **	.47 ***

* *p* < .05; ** *p* < .01; *** *p* < .001 (2-tailed).

**Table 5:** Means, standard deviations and ANCOVA results of effects of reasons for referral to CPS on event schema, narrative coherence, narrative temporal cohesion and episodic memory, controlling for children’s age.

	Disruptive Behaviour	Exposure to Domestic Violence	Neglect and Abuse	*F* (df)	*η_p_* ^2^	Power
	*M (SD)*	*M (SD)*	*M (SD)*			
Event schema	4.89 (0.63)	4.48 (0.59)	5.32 (0.53)	0.59 (2,50)	.02	.14
Narrative coherence	5.25 (0.48)	4.54 (0.45)	4.99 (0.40)	0.58 (2,50)	.02	.14
Narrative temporal cohesion	2.87 (0.35)	1.96 (0.33)	2.38 (0.29)	1.72 (2,50)	.06	.34
Episodic memory	3.75 (0.25)	2.64 (0.24)	2.39 (0.20)	8.80 (2,52) ***	.25	.96

Each model includes children’s age as a covariate. *** *p* < .001 (2-tailed).

## Data Availability

Data supporting reported results are available at https://osf.io/k9ja7 (accessed on 25 September 2021).
